# Rab6 and Rab11 Regulate *Chlamydia trachomatis* Development and Golgin-84-Dependent Golgi Fragmentation

**DOI:** 10.1371/journal.ppat.1000615

**Published:** 2009-10-09

**Authors:** Anette Rejman Lipinski, Julia Heymann, Charlotte Meissner, Alexander Karlas, Volker Brinkmann, Thomas F. Meyer, Dagmar Heuer

**Affiliations:** 1 Department of Molecular Biology, Max Planck Institute for Infection Biology, Berlin, Germany; 2 Microscopy Core Facility, Max Planck Institute for Infection Biology, Berlin, Germany; Duke University Medical Center, United States of America

## Abstract

Many intracellular pathogens that replicate in special membrane bound compartments exploit cellular trafficking pathways by targeting small GTPases, including Rab proteins. Members of the Chlamydiaceae recruit a subset of Rab proteins to their inclusions, but the significance of these interactions is uncertain. Using RNA interference, we identified Rab6 and Rab11 as important regulators of *Chlamydia* infections. Depletion of either Rab6 or Rab11, but not the other Rab proteins tested, decreased the formation of infectious particles. We further examined the interplay between these Rab proteins and the Golgi matrix components golgin-84 and p115 with regard to *Chlamydia*-induced Golgi fragmentation. Silencing of the Rab proteins blocked *Chlamydia*-induced and golgin-84 knockdown-stimulated Golgi disruption, whereas Golgi fragmentation was unaffected in p115 depleted cells. Interestingly, p115-induced Golgi fragmentation could rescue *Chlamydia* propagation in Rab6 and Rab11 knockdown cells. Furthermore, transport of nutrients to *Chlamydia*, as monitored by BODIPY-Ceramide, was inhibited by Rab6 and Rab11 knockdown. Taken together, our results demonstrate that Rab6 and Rab11 are key regulators of Golgi stability and further support the notion that *Chlamydia* subverts Golgi structure to enhance its intracellular development.

## Author Summary

*Chlamydia trachomatis* is the leading cause of sexually transmitted disease and preventable blindness in humans. These bacteria survive and replicate within a membrane bound niche inside host cells, termed the inclusion. The inclusion expands as *Chlamydia* replicate, likely fuelled by the interception of cellular trafficking pathways to acquire essential nutrients. Certain host proteins, the Rab GTPases, have previously been observed at inclusion membranes, but the function of this host–pathogen interaction remains unknown. Here, we show distinct Rab proteins are important for the outcome of an infection. Depletion of Rab6 and Rab11 by RNA interference inhibited *Chlamydia*-induced fragmentation of the Golgi apparatus (GA), a key organelle functioning in the processing and packaging of cell synthesized macromolecules, accompanied by a decrease in bacterial progeny and a reduction in lipid transport to the inclusion. Our investigation of the cross-talk between *Chlamydia* and a host cell trafficking pathway not only further strengthens our group's previous finding that *Chlamydia* subverts GA structure to enhance replication, but has identified Rab6 and Rab11 as key regulators of GA stability. In the wider context, our results also raise the provocative notion of a novel anti-chlamydial treatment based on interference with GA function.

## Introduction

With an estimated 90 million new infections per year, *Chlamydia trachomatis* is the most frequently sexually transmitted bacterial species. Infections with the serovars D–K can cause pelvic inflammatory disease and inflammation of the endometrium and urethra [1],[2]. Chronic or recurring infections with *C. trachomatis* are considered to cause infertility in women. *C. trachomatis* serovars A–C infect eyes and, in cases of repeated infection, can result in scarring of the cornea leading to blindness [3]. *Chlamydia* has a unique cycle of development that alternates between two distinct bacterial forms: infectious, metabolically inert elementary bodies (EBs) and non-infectious but metabolically active reticulate bodies (RBs) [4]. Once inside host cells, *Chlamydia* remodel host cell components to fashion a membrane bound niche, termed the inclusion, via the secretion of bacterial proteins [5],[6]. This special niche is distinct from the lysosomal degradation pathway but allows interactions with the Golgi apparatus (GA), multivesicular bodies and lipid droplets [7]–[10]. Uptake of different lipids including sphingolipids and phospholipids is known to be essential for *Chlamydia* development [11]–[13]; however, the role of host proteins in this process is still largely unknown.

In eukaryotic cells the GA is the key organelle of the secretory pathway [14],[15]. The GA is comprised of numerous stacks; each consisting of four to eight membrane-bound cisternae with a cis-to-trans polarity. Newly synthesised proteins and lipids are transported from the endoplasmic reticulum (ER) via the ER-Golgi intermediate compartment (ERGIC) to the cis-Golgi. The cargo is processed while passing the GA, and mature proteins are transported from the trans-Golgi network to their specific destinations inside and outside of the cell. In mammals, several Golgi stacks are laterally linked to form a Golgi ribbon [16]. Maintenance of Golgi polarity and the Golgi ribbon is essential for correct processing of proteins and impacts sphingomyelin synthesis [17].

Several proteins are involved in the organization of the Golgi structure. The golgins, a family of coiled-coil Golgi localized proteins including golgin-84, p115 and golgin-245, are known to be essential for GA structure [18]–[20]. They are involved in constructing a proteinaceous scaffold, the Golgi matrix (GM) [21],[22]. The GM was first discovered in cells extracted from lipids and as remnants of brefeldin A (BFA)-treated cells [23]. Besides the GM, the structure of the GA depends on the action of different small GTPases including Arf1 and Sar1 [23]–[25]. Recent data suggest a direct interaction between GM proteins and activation of distinct small GTPases such as Arf1 and Rab1, which regulate Golgi homeostasis; however, the mechanisms of GA ribbon formation and Golgi homeostasis are still not fully understood [26].

We have recently shown that in *Chlamydia*-infected cells the GA is fragmented and aligned around the inclusion [27]. Fragmentation of the GA enhances the formation of infectious bacteria, whereas inhibition of Golgi fragmentation decreases numbers of infectious bacteria. In addition to the close association of Golgi ministacks with the chlamydial inclusion, Rab proteins are also recruited to the inclusion. In a study performed by Scidmore and co-workers, GFP-tagged Rab6 and Rab11 were shown to associate with *C. trachomatis* [28]. Detailed analysis demonstrated that *Chlamydia* expresses and secretes specific Rab binding proteins into the inclusion membrane, but the precise function of Rab interactions for *Chlamydia* remains elusive [29],[30].

Here we show that Rab6A and Rab11A regulate *Chlamydia* development. Loss of either Rab6A or Rab11A blocked *Chlamydia*-induced Golgi fragmentation, accompanied by a decrease in bacterial progeny and a reduction in lipid transport.

## Results

### RNAi screen identifies Rab6 and Rab11 as regulators of *C. trachomatis* infection

**Figure 1:**
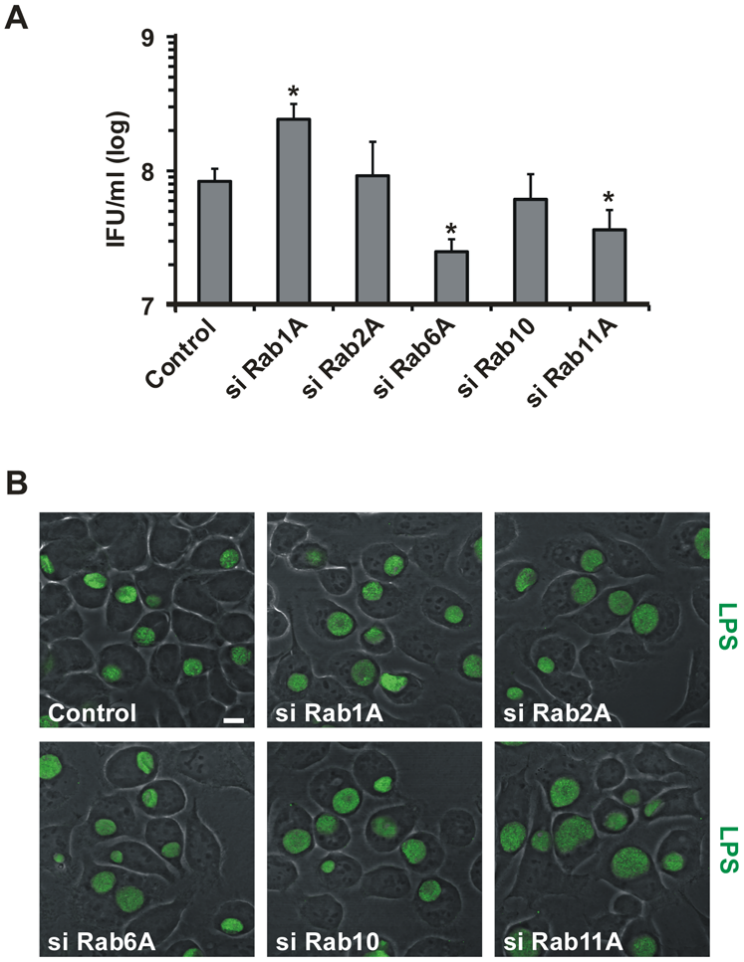
Rab6A and Rab11A are important factors for *Chlamydia* development. A) Selected Rab proteins were transiently silenced by transfection of specific siRNAs. At 3 d *p.t.*, cells were infected with *C. trachomatis* (MOI 3) and at 44 h *p.i.* cells were lysed and infectious bacteria titrated on HeLa cells. Numbers of infectious bacteria in KD cells are expressed as log IFU (inclusion forming units)/ml. siRNA against Luciferase was used as a control. Values were obtained from three independent experiments and are mean±SE. Student's t-test was used to determine p-value,*<0.05. B) Transient Rab KD cells were infected with *C. trachomatis* (MOI 1) for 24 h. Cells were then fixed and stained with an antibody specific for chlamydial LPS. Samples were analysed at a Laser scanning confocal microscope (LSCM). Overlay of LPS staining (green) and phase contrast are shown. Images are representative of n = 3. Scale bar, 10 µm.

EGFP tagged Rab1, Rab6 and Rab11 have previously been shown to associate with the *C. trachomatis* inclusion, whereas Rab5 and Rab10 are not recruited to the inclusion [28]. Therefore, we assessed the impact of these Rab proteins on *C. trachomatis* replication by RNA interference (RNAi) *loss-of-function* analysis. siRNAs used were specific for distinct Rab isoforms (see Material and Methods). The two isoforms of Rab6A, Rab6A and Rab6A', were silenced as it has been shown that knockdown of both Rab6A and A′ delays Golgi-to-ER recycling and induces aberrant Golgi morphology [31]. For simplicity reasons we refer to KD of Rab6A and Rab6A' as Rab6A KD, unless otherwise stated. Since Rab2A controls membrane trafficking in the secretory pathway [32],[33], the function of Rab2A during *Chlamydia* infections was also addressed in our study. HeLa cells were transfected with chemically synthesized short interfering RNAs (siRNAs) and then infected with *C. trachomatis* at 3 d post transfection (*p.t.*). Two days later, cells were lysed and infectious particles were titrated on fresh HeLa cells. Numbers of infectious particles were significantly reduced after knockdown (KD) of Rab6A and Rab11A, whereas twice the amount of infectious particles was recovered from Rab1A KD cells in comparison to control cells (Figure 1A). KD of Rab2A, Rab5A and Rab10 did not significantly influence the number of infectious particles (Figure 1A, data not shown). The siRNAs used showed a KD efficiency of at least 70% compared to control cells, as analyzed by real time PCR 2 d *p.t.* (Figure S1A). Furthermore, KD of selected Rab proteins, i.e. Rab6 and Rab11, was confirmed 3 d *p.t.* by immunoblotting (Figure S1B). Interestingly, the number and size of inclusions in KD cells were almost unaffected (Figure 1B). KD of Rab6A and Rab11A in HEp-2 cells also reduced bacterial progeny compared to control cells, demonstrating that Rab6A and Rab11A are important for development of *C. trachomatis* in at least two different epithelial cell lines (Figure S1C). Taken together, these data demonstrate that Rab proteins exert distinct effects on *Chlamydia* growth and identify Rab6A and Rab11A as important factors for efficient chlamydial development.

### *Chlamydia* redifferentiation is impeded in stable Rab6A or Rab11A KD cells

**Figure 2:**
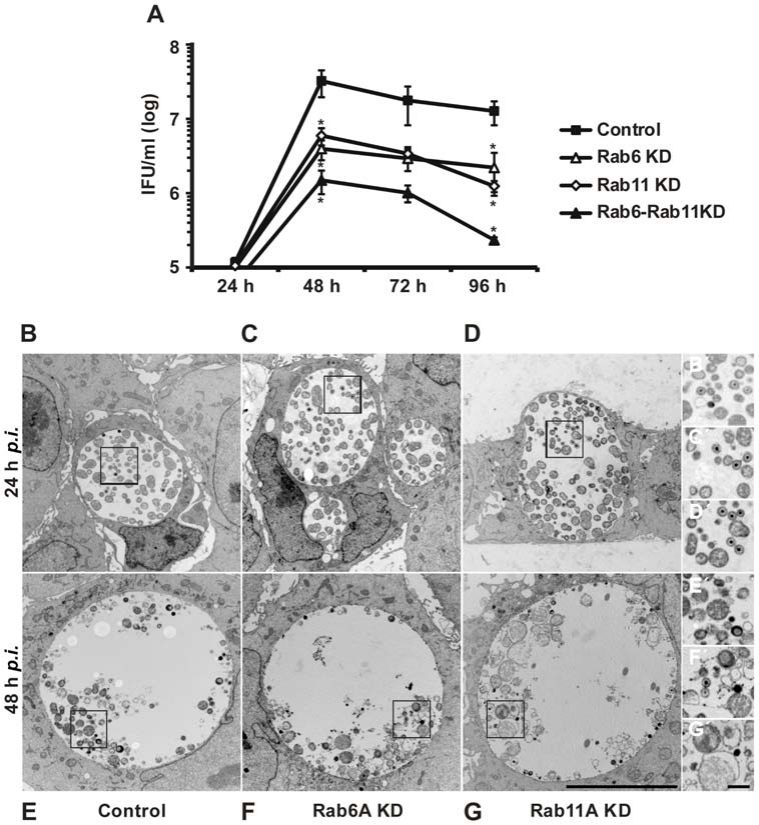
Less infectious bacteria are recovered from stable Rab6A and Rab11A KD cells compared to control KD cells. A) Control, Rab6A (Rab6A KD) and Rab11A (Rab11A KD) stable KD cells were infected with *C. trachomatis* (MOI 1) for 24 h, 48 h, 72 h and 96 h, then infectious bacteria were titrated on HeLa cells. Numbers of infectious bacteria in KD cells were expressed as log IFU (inclusion forming units)/ml. shRNA against Luciferase was used as a control. Values were obtained from three independent experiments and are mean±SE. Student's t-test was used to determine p-value, **<0.01. (B–G) Electron microscopy pictures of inclusions at 24 h *p.i.* (B–D) and 48 h *p.i* (E–G) from B,E) Control, C,F) Rab6A KD and D,G) Rab11A KD cells. (n = 3). Scale bar, 10 µm. Images B′–G′ show an enlargement of inclusions, indicated by black frames, in B,E) Control, C,F) Rab6A KD and D,G) Rab11A KD cells. Scale bar, 1 µm.

To analyze the function of Rab6A and Rab11A in *Chlamydia* infections in detail, stable KD cells were generated. HeLa cells were infected with lentiviruses expressing short hairpin RNAs (shRNAs) targeting Rab6A, Rab11A, and luciferase as a control. GFP was co-expressed for selection of lentiviral-infected cells. GFP-positive cells were sorted by FACS and analysed for loss of Rab6A and Rab11A by immunoblotting (Figure S2A). In contrast to control cells, almost no Rab6 or Rab11 was detected in the cloned KD cells (Figure S2A). Stable KD of Rab6A or Rab11A did not affect cell viability, as determined by a WST-1 assay (Figure S2B). *Chlamydia* growth was analyzed in these cells by titration of infectious progeny at 1 d to 4 d post infection (*p.i*). At 2 d *p.i.*, the number of infectious particles was significantly reduced in Rab6A and Rab11A KD cells (Figure 2A). Numbers of infectious particles peaked at 2 d *p.i.* then declined until 4 d *p.i*, indicating the cycle of development was normal albeit reduced in numbers of EBs (Figure 2A). The primary infection, as analysed by number and size of inclusions formed, in KD cells (Figure S2C and Figure S2D) was not affected, indicating that the early phase of infection was not influenced by the expressed shRNA. Interestingly, simultaneous depletion of Rab6A and Rab11A further decreased numbers of infectious particles, supporting the notion that Rab6A and Rab11A KD has an additive effect on *Chlamydia* propagation (Figure 2A). These data further support the role of Rab6A and Rab11A in *Chlamydia* development.

The two chlamydial developmental forms (EBs and RBs) differ in their appearance in transmission electron microscopy (TEM): EBs are small (∼300 nm) and electron-dense, whereas RBs are larger forms (∼1 µm) and less electron-dense. EM studies on infected stable KD cells revealed a normal chlamydial developmental cycle in control cells, characterized by high numbers of RBs in inclusions at 24 h *p.i.* and mainly EBs at 48 h *p.i.* (Figure 2B, C, D, E, F, G and Figure S2E). At 24 h *p.i.*, differences in bacterial forms per inclusion between Rab6A (28±1) and Rab11A KD (29±7) cells and control cells (30±5) were negligible, with mainly RBs detected within inclusions (Figure 2B, C, D, B′–D′ and Figure S2E). In contrast, at 48 h *p.i.* significantly less EBs were detected in Rab6A and Rab11A KD cells in comparison to control cells, even though the number of bacterial forms per inclusion in Rab6A (39±1), Rab11A KD (31±6) cells and control cells (40±8 ) was not significantly changed (Figure 2E, F, G and Figure S2E). Furthermore, in Rab6A and Rab11A KD cells membrane blebs and bacterial ghosts were also detected, indicative of defective development (Figure 2E′–G′). Taken together, these data suggest that the presence of Rab6A and Rab11A in infected cells is important for formation of infectious EBs.

### *Chlamydia*-induced Golgi fragmentation is inhibited in Rab6A or Rab11A KD cells

**Figure 3:**
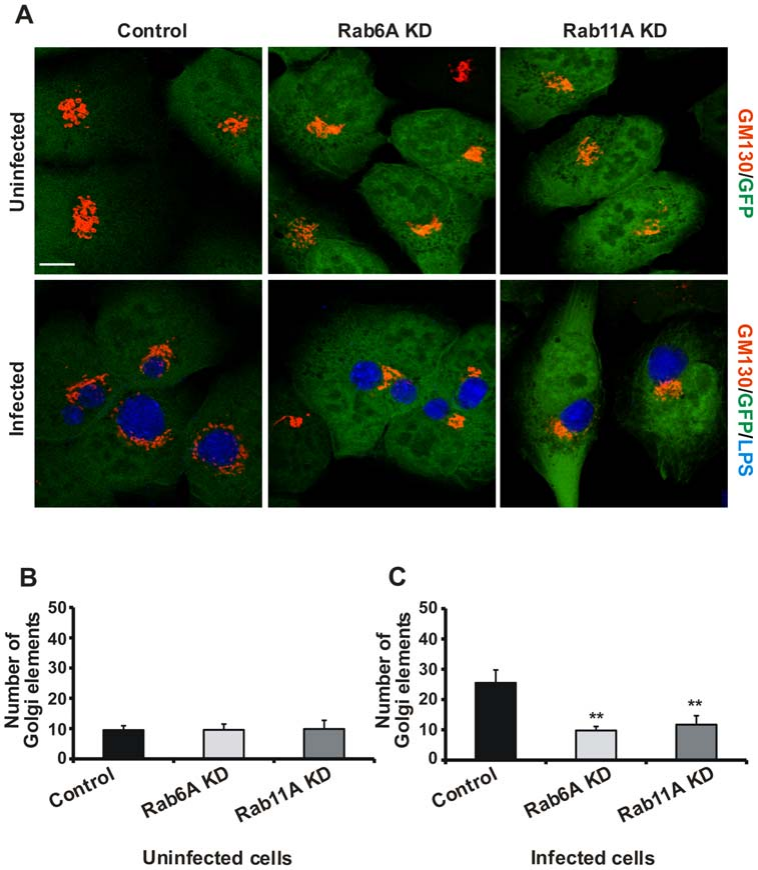
GA is not fragmented in *C. trachomatis*-infected Rab6A and Rab11A KD cells. A) Control, Rab6A KD and Rab11A KD cells were either infected with *C. trachomatis* (MOI 1) or mock-infected (uninfected). At 24 h *p.i.*, cells were fixed and stained with antibodies specific for GM130 (red channel) and LPS (blue channel). Stable KD cells express GFP as a marker of shRNA expression. shRNA against Luciferase was used as a control. Samples were analysed at a LSCM. Merge pictures are shown. Images are representative of n = 3. Scale bar, 10 µm. B) Quantification of Golgi elements in uninfected cells. Absolute numbers of Golgi elements/cell are shown. C) Quantification of Golgi elements in *C. trachomatis*-infected cells. Absolute numbers of Golgi elements/cell are shown. Values were obtained from three independent experiments and are mean±SE. Student's t-test was used to determine p-value, **<0.01.

We demonstrated previously that the GA is fragmented in *Chlamydia*-infected cells and that this enhances *Chlamydia* replication [27]. Therefore, we investigated whether Rab6A and Rab11A depletion interferes with *Chlamydia*-induced Golgi fragmentation and subsequent reduction of infectious particles. To visualize the GA in uninfected and infected KD cells, cells were immunostained with GM130-specific antibodies (Figure 3A). Depletion of Rab6A and Rab11A did not change GA structure in uninfected cells: In all cells a compact Golgi structure was detected (Figure 3A). Indeed, the GA topology in KD cells appeared even more compact in comparison to control cells. In contrast, the GA was fragmented into smaller Golgi structures that surrounded the chlamydial inclusions in infected control cells (Figure 3A). In Rab6A and Rab11A depleted cells, however, *Chlamydia*-induced Golgi fragmentation was blocked. A compact Golgi structure was observed close to the inclusions (Figure 3A), and numbers of Golgi elements did not increase (Figure 3B and C). Furthermore, in cells transiently transfected with siRNAs for Rab6A and Rab11A, normal GA structure was not affected and, most importantly, *Chlamydia*-induced Golgi fragmentation was inhibited (Figure S3A). To test if Golgi fragmentation in infected Rab6A and Rab11A KD cells could be rescued by ectopic expression of the respective Rab protein, we overexpressed myc∶Rab6A or eGFP-Rab11A. These cells were then infected with *C. trachomatis* and analysed for Golgi fragmentation by staining of the Golgi marker, GM130 or Giantin, respectively. Expression of the corresponding Rab protein in the respective cell lines restored the ability of *Chlamydia* to fragment the GA, as indicated by the small GM130- and Giantin-positive elements surrounding inclusions in either Rab6A or Rab11A expressing stable KD cells (Figure S3B and S3C). Although we cannot exclude a role for Rab6A' in *C. trachomatis* Golgi fragmentation, the ability of Rab6A to rescue *Chlamydia*-induced Golgi fragmentation in Rab6A stable KD cells suggests that Rab6A plays a pivotal role (Figure S3B). Interestingly, silencing of Rab1A, which increased number of infectious particles, induced Golgi fragmentation in control cells (Figure S3D).

Our previous work showed that Golgi fragmentation by *C. trachomatis* is accompanied by processing of the host Golgi matrix protein, golgin-84 [27]. To address if Rab6A or Rab11A KD interfere with *Chlamydia*-induced Golgi fragmentation by inhibition of golgin-84 processing, we assessed the amount and size of this protein by immunoblotting of lysates obtained from infected stable KD cells. Golgin-84 was still processed in infected Rab6A and Rab11A stable KD cells (Figure S3E), suggesting that Rab6A and Rab11A control Golgi fragmentation downstream of golgin-84.

Taken together, our data show Rab6A and Rab11A are essential for *Chlamydia*-induced Golgi fragmentation and further strengthens the hypothesis that Golgi fragmentation can influence the outcome of *Chlamydia* infections.

### Rab6A or Rab11A KD blocks golgin-84-dependent Golgi fragmentation in non-infected cells

**Figure 4:**
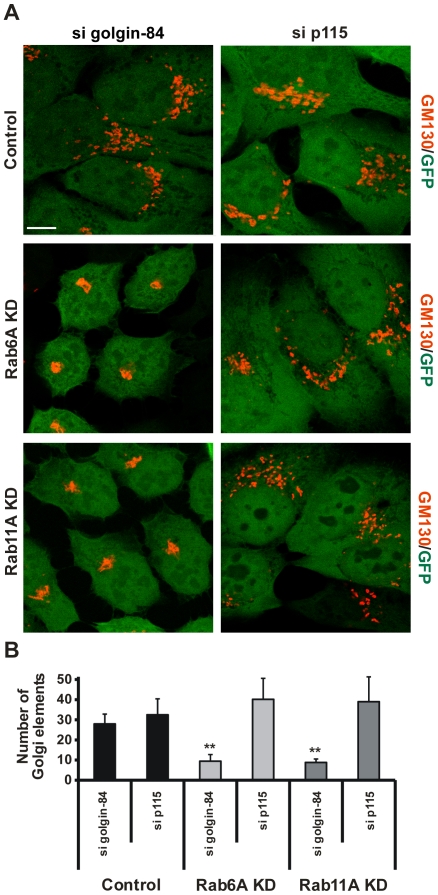
Silencing of Rab6A and Rab11A expression suppresses golgin-84-dependent Golgi fragmentation. A) Control, Rab6A KD and Rab11A KD cells were transiently silenced for the expression of golgin-84 and p115 by siRNA transfection. At 4 d *p.t.*, cells were fixed and stained for the Golgi marker GM130 (red channel). Stable KD cells express GFP as a marker of shRNA expression. shRNA against Luciferase was used as a control. Samples were analysed at a LSCM. Overlay of GM130 and GFP are shown. Images are representative of n = 3. Scale bar, 10 µm. B) Quantification of Golgi elements in the different double KD cells. Absolute numbers of Golgi elements/cell under the different experimental conditions are depicted. Values were obtained from three independent experiments and are mean±SE. Student's t-test was used to determine p-value, **<0.01.

To test the interdependence of golgin-84, Rab6A and Rab11A in Golgi fragmentation, we performed double KD experiments. Rab6A and Rab11A KD cells were transiently transfected with siRNAs for either golgin-84 or p115. Similar to golgin-84, p115 is a Golgi matrix protein and depletion of p115 has been shown to induce Golgi fragmentation [20]. The structure of the GA in the different double KD cells was visualized by GM130 staining 4 d *p.t.* As previously described, KD of golgin-84 and p115 in control cells fragmented the GA into smaller Golgi elements (Figure 4A; [27]). In contrast, a compact Golgi structure was detected in Rab6A and Rab11A double KD cells following depletion of golgin-84 (Figure 4A). Efficiency of golgin-84 KD was not affected by depletion of Rab6A or Rab11A (Figure S4). Golgi fragmentation upon depletion of p115, however, was insensitive to KD of Rab6 and Rab11 (Figure 4A). These observations were confirmed by quantification of Golgi fragmentation (Figure 4B). Thus, our findings demonstrate Golgi fragmentation by depletion of golgin-84 requires Rab6A and Rab11A, whereas p115-mediated Golgi fragmentation is independent of Rab6 and Rab11.

### Golgi fragmentation by p115 depletion rescues *Chlamydia* development in Rab6A or Rab11A KD cells

**Figure 5:**
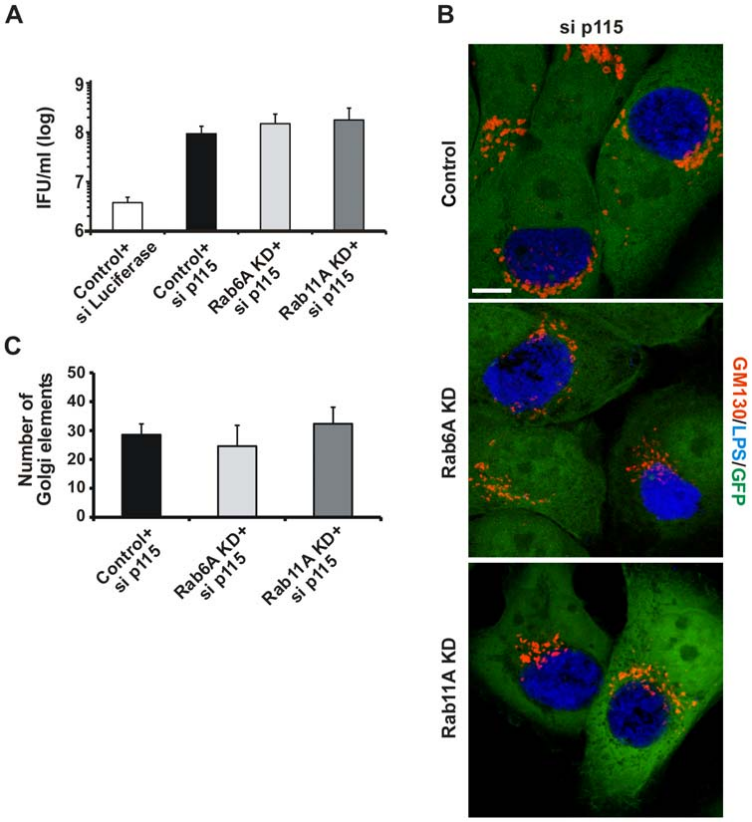
Loss of p115 induces Golgi fragmentation in infected cells independently of Rab6A and Rab11A. A) Control, Rab6A KD and Rab11A KD cells were transiently silenced for the expression of p115 by siRNA transfection. Control cells were additionally transfected with an siRNA targeting Luciferase. At 3 d *p.t.* cells were infected with *C. trachomatis* (MOI 3). At 44 h *p.i.*, cells were lysed and infectious bacteria were titrated on HeLa cells. IFU/ml expressed in log scale B) Control, Rab6A KD and Rab11A KD depleted cells were infected with *C. trachomatis* at an MOI 1. Infected cells were fixed and stained for GM130 (red channel) and chlamydial LPS (blue channel). shRNA against Luciferase was used as a control. Stable KD cells express GFP as a marker of shRNA expression. Samples were analysed at a LSCM. Merged pictures are shown. Images are representative of n = 3. Scale bar, 10 µm. C) Quantification of Golgi fragmentation. Absolute numbers of Golgi elements/cell are depicted. (A, C) Values were obtained from three independent experiments and are mean±SE.

Next, we asked if Golgi fragmentation by p115 depletion could rescue *Chlamydia* development in Rab6A and Rab11A KD cells. Firstly, p115 expression was transiently silenced in Rab6A and Rab11A KD cells, then cells were infected and numbers of infectious progeny determined at 2 d *p.i.* Depletion of p115 in control cells dramatically increased (by more than one log) chlamydial progeny, in comparison to control shRNA cell lines transfected with siRNAs for luciferase (Figure 5A). This is consistent with our previous observations showing increased *Chlamydia* progeny in cells displaying a fragmented GA [27]. Interestingly, transfection of Rab6A and Rab11A KD cells with a p115-specific siRNA, rescued bacterial development in these otherwise less supportive cell lines (Figure 5A). The increase in numbers of infectious bacteria correlated well with changes in the GA structure after depletion of p115 in Rab6A and Rab11A KD cells (Figure 4B). Accordingly, in infected cells Golgi fragmentation was detected irrespective of Rab6A or Rab11A KD (Figure 5B). Quantification of Golgi elements confirmed Golgi fragmentation in these infected KD cells (Figure 5C). These observations demonstrate that reduced chlamydial development in Rab6A and Rab11A KD cells can be circumvented by Golgi fragmentation mediated by p115 down-regulation.

### Rab6A or Rab11A KD reduces transport of sphingolipids to the inclusion

**Figure 6:**
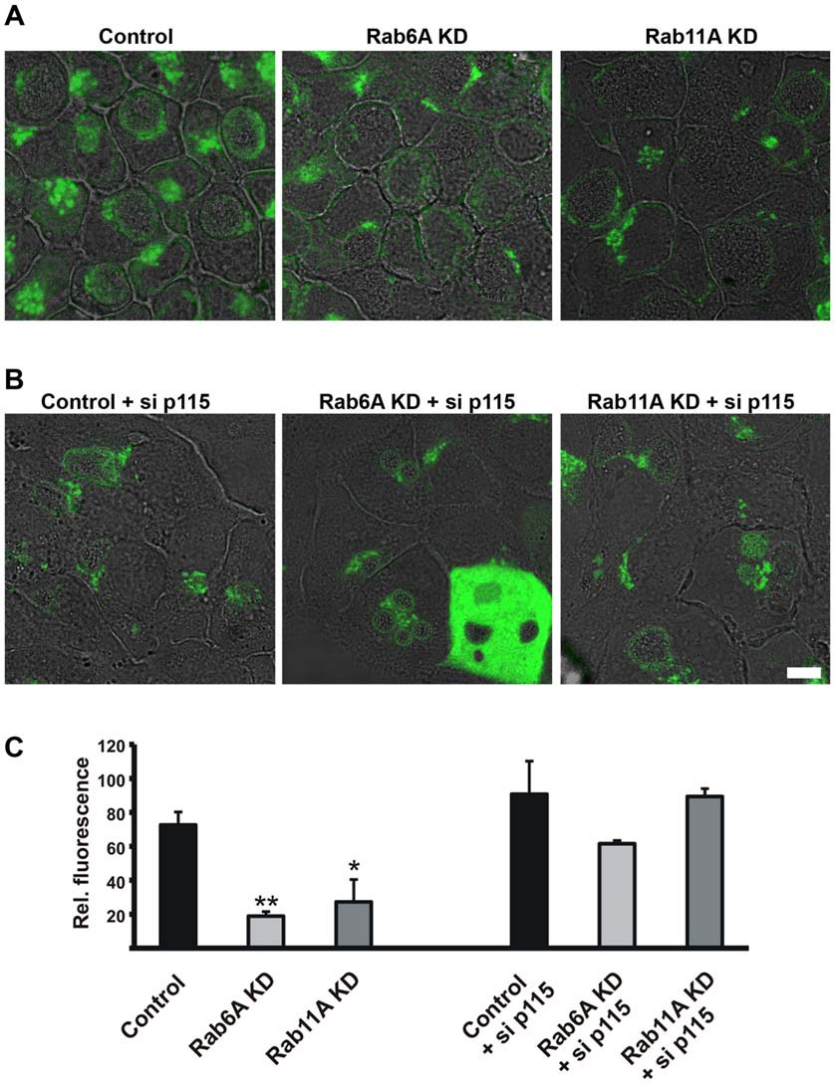
Efficient sphingolipid transport to *Chlamydia* depends on Rab6A and Rab11A expression. A) Control, Rab6A KD and Rab11A KD cells were infected with *C. trachomatis* (MOI 1). At 1 d *p.i.*, cells were labeled with BODIPY-FL-Ceramide (green channel) and lipid transport to the inclusion was analysed for about 60 min by live-cell microscopy. Images represent acquisition at the 48 min time point. Inclusions are seen as black holes as GFP does not cross inclusion membranes. B) Control, Rab6A KD and Rab11A KD cells were transiently transfected with siRNA against p115 and at 3 d *p.t.* infected with *C. trachomatis* (MOI 1). At 1 d *p.i.*, cells were labeled with BODIPY-FL-Ceramide (green channel) and lipid transport to the inclusion was analysed for about 60 min by live-cell microscopy. Images represent acquisition at the 48 min time point. Inclusions are seen as black holes as GFP does not cross inclusion membranes. Images are representative of five independent experiments. Scale bar, 10 µm. C) Quantification of lipid transport in Control, Rab6A and Rab11A KD cells and in cells simultaneously silenced for the Control and p115, or Rab6 and p115, or Rab11 and p115. Diagram shows the fluorescence intensities of the inclusions relative to that of the Golgi apparatus at 48 min. Values were obtained from three inclusions and three Golgi areas and are mean±SD. Student's t-test was used to determine p-value, *<0.05, **<0.01.

Chlamydiaceae acquire sphingolipids from the host cell [11]–[13]. Therefore, we investigated whether Rab6A and Rab11A KD interfere with bacterial sphingolipid uptake. The transport of BODIPY-FL labeled ceramide to the inclusions was followed using live-cell microscopy (Figure 6A, Videos S1, S2, S3). Labeled lipids were rapidly transported to bacterial inclusions within 10 min of lipid addition and incorporated into bacteria after 20–30 min. In contrast, transport of labeled ceramide was reduced in Rab6A and Rab11A KD cells infected with *C. trachomatis*. Thirty minutes after lipid addition, inclusions were only slightly stained, in a rim-like pattern, with most of the lipid accumulating in a structure close to the inclusions that resembled the GA. Similar results were obtained with a generated stable cell line silenced simultaneously for Rab6A and Rab11A (Video S4).

The observed Golgi fragmentation in infected cells has been linked to boosted lipid acquisition by *Chlamydia* [27]. To test if fragmentation of the GA by depletion of p115 could overcome inhibition of sphingolipid transport in Rab6A and Rab11A KD cell lines, trafficking of BODIPY-FL labeled ceramide was analysed in cells simultaneously silenced for the control and p115 or Rab11A and p115 or Rab6A and p115 (Figure 6B, Videos S5, S6, S7). As hypothesized, fragmentation by loss of p115 increased acquisition of sphingolipid by *Chlamydia*, as seen by a clear staining of inclusions in comparison to single Rab6A or Rab11A KD cell lines (Figure 6A and 6B). Quantification of sphingolipid transport into inclusions confirmed the live-cell data. In inclusions of Rab6A and Rab11A KD cells, significantly less relative fluorescence intensity was detected in comparison to control cells (Figure 6C). In KD cells transiently silenced for p115, the relative fluorescence intensities of inclusions were increased in comparison to inclusions within the Rab6A and Rab11A KD cells (Figure 6C). These data demonstrate that Rab6A and Rab11A are important for efficient sphingolipid transport to *Chlamydia* and further support the notion that Golgi fragmentation can boost chlamydial uptake of sphingolipid in the absence of Rab6A and Rab11A.

## Discussion

Here we demonstrate the biological role of distinct Rab proteins in *C. trachomatis* infection. Efficient *C. trachomatis* replication and development was previously found to be dependent on the fragmentation of the Golgi apparatus [27]. Using a *loss-of-function* approach we now show a regulatory role of the small GTPases Rab6A and Rab11A in modulating Golgi morphology of infected cells, thus influencing chlamydial development.

Intracellular bacteria residing inside a membrane-bound compartment such as members of the Chlamydiaceae, *Salmonella spp*. and *Legionella pneumophila*, are known to interact with cellular proteins belonging to the family of small GTPases including Rab proteins [34]. Previous studies using GFP-Rab fusion proteins demonstrated an orchestrated recruitment of distinct Rab proteins to *C. trachomatis* inclusions: Rab1, Rab4, Rab6 and Rab11 interacted with *C. trachomatis* inclusions, whereas GFP-Rab5 did not [28]. Furthermore, GFP-Rab10 was shown to colocalize with *C. pneumoniae* inclusions but not with inclusions of *C. trachomatis* [28]. However, the functional significance of this recruitment was unknown. These interactions are considered to be extremely important for *Chlamydia* spp. as they only replicate inside a membrane bound compartment, which interacts with cellular trafficking pathways to acquire essential nutrients. After invasion of the host cell, *Chlamydia* actively remodels its vacuole to avoid fusion with lysosomes [8], [9], [35]–[37]. This early remodeling is characterized by secretion of bacterial proteins such as Ct229, which has been shown to interact with Rab4 [30]. Rab11, another Rab specific for recycling endosomes, was detected on the nascent vacuole as early as 1 h *p.i.*, whereas other early endosomal and lysosomal makers, including early endosomal antigen 1 (EEA1), Rab5 and Lamp1, were absent from inclusions [8], [9], [28], [35]–[37]. Rab4 and Rab11 together with Rab5 sequentially regulate recycling of receptors such as Transferrin receptor, which partially colocalizes with Rab11 at the inclusion [28]. Knockdown of Rab5A, which is not recruited to the *C. trachomatis* inclusion, did not alter *C. trachomatis* growth, demonstrating that this protein is not functionally involved in *C. trachomatis*-host interactions. This further strengthens the existing observations that *Chlamydia* does not directly depend on the early endosomal trafficking pathways [8], [9], [35]–[37]. Furthermore, it is interesting to note that neither Rab4A nor Rab11A KD reduced inclusion formation, suggesting that the entry and inclusion formation is independent of Rab4A or Rab11A. These data are consistent with the view that *Chlamydia* can enter nonprofessional phagocytes by different mechanisms [38].

Another hallmark of *Chlamydia* infections is the transport of the early inclusion towards the microtuble-organization centre (MTOC) [39]. Since Rab11A is recruited to chlamydial vacuoles as early as 1 h *p.i.* and is known to control endosome to trans-Golgi transport [28], it is a likely candidate to control this process. Interestingly, the formation of inclusions in Rab6A and Rab11A KD cells was not affected. Silencing of Rab6A and Rab11A decreased the formation of EBs at later time points of chlamydial development, indicating that Rab6A and Rab11A are involved in later processes. Our data show that Rab6A and Rab11A are dispensable for the establishment of the inclusion, but are important for the completion of the chlamydial cycle of development. A recent RNAi-based *loss-of-function* screen in Drosophila S2 cells highlighted the importance of Rab proteins during *Chlamydia* infection [40]. Consistent with our results, Rab6 and Rab11 did not influence the establishment of an infection in S2 cells. Unfortunately, the formation of infectious particles could not be addressed due to a general impairment of chlamydial development in this model.

We previously demonstrated that fragmentation of the GA could enhance chlamydial propagation, whereas inhibition of Golgi fragmentation dramatically reduced the formation of infectious bacteria [27]. Here we show that *Chlamydia*-dependent Golgi fragmentation was blocked in Rab6A and Rab11A KD cells and formation of infectious particles was decreased; but how could these Rab proteins actually regulate Golgi structure? The structure of the GA is a balance between transport and adherence of stacks, which can be regulated by small GTPases. Furthermore, proteins of the GM stabilize Golgi structure. Depletion of golgins that belong to the GM including golgin-84, p115 and giantin, amongst others, induced Golgi fragmentation and enhanced bacterial development. Recently, an essential role of Rab proteins in maintaining Golgi trafficking and structure by directly regulating golgins was described [41]. Our study shows that depletion of Rab6 and Rab11 suppressed fragmentation of the GA in golgin-84-depleted cells and *Chlamydia*-infected cells. These data suggest Rab6A and Rab11A act in a pathway with golgin-84 and regulate either trafficking of vesicles and/or adherence of Golgi stacks by golgin-84. It is tempting to speculate that Rab6A and Rab11A positive vesicles directly transport nutrients from the GA to *Chlamydia* inside the inclusion. This view is supported by the observation of Rab6A and Rab11A recruitment to the inclusions, and by the reduced transport of sphingolipids to bacteria in Rab KD cells. However, previous work demonstrating that Rab6 can be recruited to the inclusion independently of the GA contradicts this model [42]. Furthermore, we found that the inhibitory effect of Rab6A and Rab11A KD on *Chlamydia* propagation could be reversed by simultaneous depletion of p115, causing Golgi fragmentation regardless of Rab6A and Rab11A.

Our data argues for a model in which *Chlamydia* destabilizes the Golgi apparatus by targeting proteins that stabilize the Golgi apparatus under normal cellular conditions, such as golgin-84; and also by stimulation of Golgi destabilizing factors such as Rab6 and Rab11. In this model, Rab6 and Rab11 would regulate transport of lipids to *Chlamydia* and affect Golgi structure, further enhancing the development of infectious particles (Figure S5). Probing for the functional connection between golgin-84 and these Rab proteins is a future task that requires further experimental data.

In summary, our work demonstrates that Rab6A and Rab11A influence the development of *Chlamydia* by regulating golgin-84dependent fragmentation of the GA. This further strengthens the view that the structure of the GA is an important modulator of *Chlamydia* propagation and thus suggests the intriguing possibility of an anti-chlamydial treatment based on interference with GA structure and function.

## Materials and Methods

### Cell culture

HeLa and HEp-2 cells were grown in Hepes-buffered RPMI supplemented with 10% FCS, and 293T cells in DMEM supplemented with 10% FCS, 2mM glutamine and 1mM sodium pyruvate, all at 37°C in a humidified incubator containing 5% CO_2_.

### Antibodies and plasmids

Antibodies were obtained from following sources: m-α-GM130, rb-α-Giantin, m-α-golgin-84 and m-α-Rab11 (BD Bioscience), rb-α-chlamydia LPS (Milan Analytica AG), m-α-chlamydia Hsp60 (Alexis Biochemicals), m-α-β-actin (Sigma), m-α-chlamydia MOMP (University of Washington), rb-α-Rab6 (Santa Cruz), secondary antibodies conjugated to HRP (Jackson Immuno Research Laboratories), m-α-myc (Santa Cruz), secondary antibodies labeled to Cy2, Cy3 and Cy5 (Molecular Probes). pmyc∶Rab6AWT was a kind gift from B. Goud, Institut Curie, Paris, France and peGFPRab11AWT was kindly provided by M. Zerial, MPI for Cell Biology, Dresden, Germany. The lentiviral vector pLVTHM and the packaging vectors pMD2G and psPAX2 were a generous gift from D. Trono, Lausanne, Switzerland.

### Bacteria

*Chlamydia trachomatis* serovar L2 was propagated in HeLa and HEp-2 cells in RPMI medium supplemented with 5% FCS (infection medium). For chlamydial infections, cells were washed twice with infection medium and infected with *C. trachomatis* L2 at the indicated multiplicity of infection (MOI) for 2 h at 35°C in a humidified incubator containing 5% CO_2_. Infected cells were subsequently washed and incubated at 35°C for time points as indicated.

### Infectivity assay

Cells were transfected with siRNAs specific for different Rab and Golgi proteins and Luciferase (control) for 72 h. Next, transfected cells or stable KD cells were infected with *C. trachomatis* for another 48 h, lysed with glass beads and titrated on HeLa and HEp-2 cells. After 24 h, cells were fixed, permeabilized and stained with an antibody against chlamydial Hsp60. Numbers of inclusions formed by chlamydial progeny were counted in ten fields by microscopical analysis and expressed as inclusion forming units (IFU). IFU was normalized to cell number and expressed as a percentage of the luciferase control.

### Determination of cell numbers

Cell numbers for normalization were determined by LDH release (Cytotoxicity detection kit [Roche]). Cell lysate obtained after glass bead lysis was centrifuged for 10 min at 270×g to remove debris. 100 µl of a 1∶50 dilution was transferred to a 96-well plate and 100 µl of reaction solution added. Incubation was performed under light-protected conditions for 20–30 min and stopped using 50 µl Stop solution. Absorption was measured in an ELISA photometer at 450 nm wavelength.

### Analysis of inclusions in stable KD cells

KD cells stably expressing shRNAs were infected at the indicated MOI with *C. trachomatis* for 24 h. Cells were fixed with ice-cold methanol and stained with a specific antibody against chlamydial MOMP. Additional DNA was counterstained with Hoechst reagent. Pictures were taken with an Olympus microscope using Scan^R^ software. Calculated numbers of inclusions per nuclei were depicted in bar graphs. Size of inclusion was determined as pixel area.

### Immunofluorescence and confocal microscopy

Cells were grown in plates with coverslips according to the assay, fixed with 2% PFA and permeabilized for 20 min with 0.2% BSA in PBS and 0.2% Triton X-100. Cells were then incubated for 1 h with antibodies targeting different Golgi markers or bacteria, followed by incubation with a fluorescently labeled secondary antibody. Samples were mounted in MOWIOL. Images were taken with a Leica TCS-SP confocal microscope and processed using Adobe Photoshop 11.0.

### Immunoblotting

For immunodetection, cells were lysed in RIPA buffer on ice at the indicated times. Protein concentration was determined using a bicinchoninic acid (BCA) kit (Pierce). Total proteins (20 µg) were resolved by reducing SDS-PAGE. Separated proteins were transferred onto a PVDF membrane. Different proteins were detected by incubation with specific antibodies followed by appropriate secondary antibodies and visualized using ECL reagent.

### Generation of stable cells

shRNA Rab6A (UACGGUCUUCUUUGAGGUCAA) and Rab11A (GGUUUCAGUAUGUCUGAAGAG) constructs were stably transduced into HeLa cells using a lentiviral based expression system [43]. Design of shRNAs was performed with the BLOCK-iT™ RNAi Designer from Invitrogen and cloned into the vector pLVTHM. The shRNAs used were specific for distinct Rab isoforms. Rab6A shRNA targets both Rab6A isoforms, Rab6A and Rab6A'.

293T cells were transfected using calcium phosphate transfection together with the packaging vectors psPAX2 und pMD2G. Viruses were harvested from the supernatant and used for infection of HeLa cells in the presence of polybrene (5 µg/ml). GFP-positive cells were selected at 7 d *p.i.* and sorted into 96-well plates using flow cytometry. Cells transduced with lentiviruses coding for a firefly luciferase shRNA (target sequence -AACUUACGCUGAGUACUUCGA) were used as a control.

### RNA interference

siRNAs were purchased from QIAGEN and validated at the MPI for Infection Biology, Berlin. HeLa cells were seeded into 12-well plates one day before transfection and grown to 70% confluency. Transfection was performed using QIAGEN RNAiFect according to the manufacturer's guidelines. In brief, 1 µg siRNA was added to EC-R buffer, mixed with 6 µl RNAiFect and incubated for 15 min at room temperature. The liposome/RNA mix was added to cells with 600 µl growth medium. Twenty four hours later, cells were trypsinized and seeded into new cell culture plates depending on the experiment. Cells were infected 72 h *p.t.* for the indicated time points at the indicated MOI.

### Validation of RNAi by q-PCR

siRNA validation was performed according to Machuy et al. [44]. Briefly, one day before transfection 3000 cells/well were seeded into 96-well plates. In three independent experiments siRNAs with a final concentration of 56 nM were transfected with 0.25 µl RNAiFect. Luciferase siRNA was used as a control.

The following siRNAs were designed and produced by Qiagen (target sequence in XYN19 format). The siRNAs used were specific for distinct Rab isoforms. Rab6A siRNA targets both Rab6A isoforms, Rab6A and Rab6A'. Luciferase (AACUUACGCUGAGUACUUCGA), Rab1A (GTCCAGCATGAATCCCGAATA), Rab2A (GGCGACACAGGTGTTGGTAAA), Rab5 (ATTCATGGAGACATCCGCTAA), Rab6A (CAGATTCATGTATGACAGTTT), Rab10 (ACCTGCGTCCTTTTTCGTTTT) and Rab11A (AAGAGUAAUCUCCUGUCUCGA).

The following primers were used for RT-PCR:

GAPDH forward 5′-GGTATCGTGGAAGGACTCATGAC-3′, GAPDH reverse 5′-ATGCCAGTGAGCTTCCCGTTCAG-3′, Rab1A forward 5′-TCCCGGAACAGCCTATCTCAT-3′, Rab1A reverse 5′-TCCACACCAATTGTGCTGATG-3′, Rab2A forward 5′-TCATAATCGGCGACACAGGTG-3′, Rab2A reverse 5′-AGGATTCTTGCCCTGCCGTAT-3′, Rab5 forward 5′-TGGTCAAGAACGATACCATAG-3′, Rab5 reverse 5′-ATTGTCATCTGCATAGGACTG-3′, Rab6A forward 5′-CTCCTCTAGTTCCACAATGTC-3′ Rab6A reverse 5′-TATCCCACAGCTGAAGCCTG-3′, Rab10 forward 5′-TGCTTTTCAAGCTGCTCCTGA-3′, Rab10 reverse 5′-ATGATACCCATTGCGCCTCTG-3′, Rab11A forward 5′-ACGACGAGTACGACTACCTC-3′ and Rab11A reverse 5′-TTCCATCAACCTGGATGCTTC-3′. The relative expression levels of target mRNA were normalized against control transfected cells. GAPDH was used as an internal standard.

### Transmission electron microscopy

Control, Rab6 and Rab11 KD cells were seeded into 6-well plates and infected with *C. trachomatis* for 24 h and 42 h at a MOI 1. Cells were washed with PBS and fixed in 2.5% glutaraldehyde. Samples were then incubated with 0.5% osmiumtetroxide and 2% uranylacetate, contrasted with 0.1% tannin acid, dehydrated and epoxy resin-embedded. Analysis was performed at a Leo 906E Transmission electron microscope. For quantification of bacterial forms, more than 1000 particles in more than 34 inclusions for each KD cell line were counted.

### Determination of cell viability by WST-1 assay

The viability of KD cells was determined using the WST-1 reagent (Roche). The assay was performed in triplicate using a 96-well format. The WST-1 reagent was diluted 1∶5 in cell culture medium containing 5% FBS, added directly to the cultures and incubated for 30 min at 37°C and 5% CO_2_. As a negative control, stable shRNA luciferase expressing cells were lysed by Triton X-100 before addition of the reagent. The OD_450_ was measured and absorption of control cells was set to 100%.

### Quantification of Golgi elements

Confocal images of specific samples were used to quantify Golgi fragmentation. The numbers of Golgi elements in 30 cells per experiment were counted. Therefore, a fixed threshold was applied to all images and elements were counted using the Analyse Particles function in ImageJ software. Three independent experiments were performed to analyse Golgi fragmentation.

### Live-cell microscopy

Cells were seeded onto glass-bottom dishes (MatTek Corporation) and infected with *C.trachomatis* (MOI 1). One day *p.i.* dishes were transferred to an inverted confocal microscope (SP5, Leica) equipped with an incubator heated to 37°C. Every 60 s, a confocal image was recorded for fluorescence and differential interference contrast (DIC). BODIPY-FL C5-ceramide was added directly to the cells. To better visualize ceramide uptake into cells the intensity of GFP fluorescence was reduced. For quantification of lipid transport, mean fluorescence intensities of labeled lipid were determined by defining regions of interest (ROI) that were locally stable throughout the course of the experiment, both in the inclusion and the GA. Fluorescence intensities of the inclusions were expressed relative to the GA at 48 min.

### Gene IDs for used proteins

Rab1A Gene ID 5861, Rab2A Gene ID 5862, Rab5A Gene ID 5868, Rab6A Gene ID 5870, Rab10 Gene ID 10890, Rab11A Gene ID 8766, p115 Gene ID 8615, golgin-84 Gene ID 9950.

## Supporting Information

Figure S1 Knockdown of Rab proteins. A) Analysis of KD efficiency after siRNA treatment via quantitative real time PCR at 48 h *p.t.* Diagram shows KD efficiency in percent to control cells. B) HeLa cells were transfected with siRNAs against Rab6A, Rab11A and Luciferase (control). At 3 d *p.t.*, cells were lysed in RIPA buffer and proteins were separated by SDS-PAGE. Proteins were transferred to PVDF membranes followed by immunoblot with antibodies specific for Rab6 and Rab11 and actin. Actin was used as loading control. Blots are representative of n = 3. C) Three days *p.t.* Rab6A and Rab11A siRNA-treated Hep-2 cells were infected with *C. trachomatis* (MOI 3). At 44 h *p.i.*, cells were lysed and infectious bacteria were titrated on HeLa cells. Numbers of infectious bacteria in KD cells were expressed as IFU (inclusion forming units). shRNA against Luciferase was used as a control. (A, C) Values were obtained from three independent experiments and are mean±SE. Student's t-test was performed to determine p-value, *<0.05.
(0.37 MB TIF)

Figure S2 *Chlamydia* infection in stably expressing Rab protein knockdown HeLa cells. A) Control (Luciferase), Rab6 KD and Rab11 KD cells were lysed in RIPA buffer and proteins were separated by SDS-PAGE. Proteins were transferred to PVDF membranes followed by immunoblot with antibodies specific for Rab6, Rab11 and actin. Actin was used as loading control. Blots are representative of n = 3. B) Cell viability of stable KD cells monitored using the WST-1 assay. Diagram shows percent of cell viability normalized to luciferase control cells. TritonX-100 treatment prior to analysis was used as a negative control for the assay. C and D) Quantification of formed inclusions and analysis of inclusion size in KD and control cells. Cells were infected with different MOIs as indicated. At 24 h *p.i.*, cells were fixed and stained for chlamydial MOMP. DNA was counter stained with Hoechst reagent. Samples were analysed with an automated microscope. D) Inclusion size was measured by determination of the pixel area. E) Quantitative analysis of EBs and RBs from electron micrographs. KD cells were infected with *C. trachomatis* for 24 h and 48 h. Luciferase cells were used as a control. Relative numbers of EBs and RBs, expressed as a percentage, were determined in more than 33 cells. (B–E) Values were obtained from three independent experiments and are mean±SE. Student's t-test was performed to determine p-value, *<0.05, **<0.01.
(0.59 MB TIF)

Figure S3 A) siRNA-treated cells were infected with *C. trachomatis* (MOI 1) for 24 h. Cells were then fixed and stained with an antibody specific for the Golgi protein GM130 (red channel) and chlamydial LPS (green channel). Samples were analysed at a LSCM. Overlays are shown. B) Expression of pmyc∶Rab6AWT in Rab6A KD cells. At 26 h *p.i.*, cells were fixed and stained with antibodies specific for myc (red channel) and the Golgi protein Giantin (blue channel). Samples were analysed at a LSCM. Merge picture is shown. A single image for Giantin staining is shown in gray scale. C) Expression of peGFPRab11AWT in Rab11A KD cells. At 26 h *p.i.*, cells were fixed and stained with an antibody specific for the Golgi protein GM130 (red channel). Stable KD cells express eGFP as a marker for shRNA expression, therefore only high levels of eGFP-Rab11 could be detected. Intensity of the GFP channel was reduced to better visualize eGFP-Rab11. Samples were analysed at a LSCM. Merge pictures are shown. D) Rab1A-siRNA-treated Luciferase control cells were infected with *C. trachomatis* (MOI 1) for 24 h. Cells were then fixed and stained with an antibody specific for the Golgi protein GM130 (red channel) and chlamydial LPS (blue channel). Stable KD cells express GFP as a marker of shRNA expression. Samples were analysed at a LSCM. Overlays are shown. E) Golgin-84 cleavage in KD cells. Control (Luciferase), Rab6A KD and Rab11A KD cells were lysed in RIPA buffer and proteins were separated by SDS-PAGE. Proteins were transferred to PVDF membranes followed by immunoblot with antibodies specific for golgin-84 and actin. Actin was used as loading control. Images and blots are representative of n = 3. Scale bar, 10 µm.
(3.13 MB TIF)

Figure S4 KD of golgin-84 in transiently transfected Rab6A and Rab11A KD cells. Golgin-84 KD efficiency in KD cells. Transiently transfected control (Luciferase), Rab6A and Rab11A KD cells were lysed in RIPA buffer and proteins were separated by SDS-PAGE. Proteins were transferred to PVDF membranes followed by immunoblot with antibodies specific for golgin-84 and actin. Actin was used as loading control. Blot is representative of n = 3.
(0.36 MB TIF)

Figure S5 Schematic model of Rab regulated Golgi fragmentation and *Chlamydia* development. Under normal cellular conditions Golgi matrix proteins, including golgin-84, stabilize the GA, whereas Rab6 and Rab11 stimulate GA fragmentation. Upon infection, *Chlamydia* can influence these factors, either positively or negatively, to ensure efficient development. In this model *C. trachomatis* targets golgin-84, Rab6 and/or Rab11 to destabilize Golgi structure and stimulate ceramide acquisition, thus enhancing the development of infectious particles.
(0.22 MB TIF)

Video S1 Time-lapse microscopy of sphingolipid transport in *C. trachomatis* infected KD cells. Control (Video S1), Rab6A KD (Video S2), Rab11A KD cells (Video S3), Rab6A-Rab11A double KD cells (Video S4), Control+siRNA p115 (Video S5), Rab6A KD+siRNA p115 (Video S6), Rab11A KD+siRNA p115 (Video S7) were infected with *C. trachomatis* (MOI 1) for one day. BODIPY-FL C5-ceramide was added directly to cells. The time-lapse covers about 60 min.
(1.60 MB MOV)

Video S2 (1.85 MB MOV)

Video S3 (1.21 MB MOV)

Video S4 (1.72 MB MOV)

Video S5 (1.93 MB MOV)

Video S6 (0.77 MB MOV)

Video S7 (1.54 MB MOV)
