# Supplementary figures and images for: Rab6 and Rab11 Regulate Chlamydia trachomatis Development and Golgin-84-Dependent Golgi Fragmentation
Source: PLoS Pathog. 2009 Oct 9;5(10):e1000615. doi: 10.1371/journal.ppat.1000615 (PMC2752117; doi:10.1371/journal.ppat.1000615)

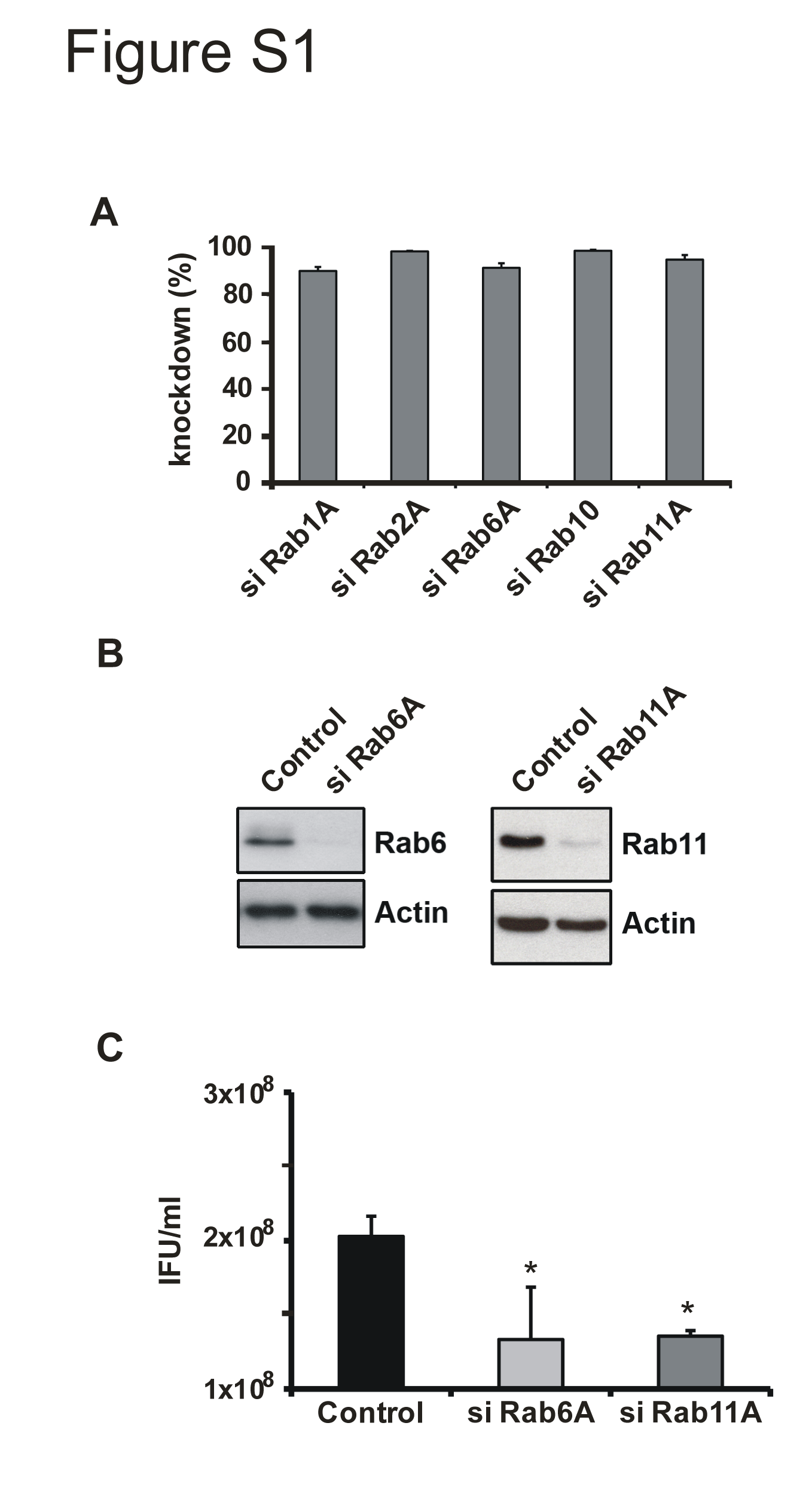

Supplement: Figure S1 — Knockdown of Rab proteins. A) Analysis of KD efficiency after siRNA treatment via quantitative real time PCR at 48 h p.t. Diagram shows KD efficiency in percent to control cells. B) HeLa cells were transfected with siRNAs against Rab6A, Rab11A and Luciferase (control). At 3 d p.t., cells were lysed in RIPA buffer and proteins were separated by SDS-PAGE. Proteins were transferred to PVDF membranes followed by immunoblot with antibodies specific for Rab6 and Rab11 and actin. Actin was used as loading control. Blots are representative of n = 3. C) Three days p.t. Rab6A and Rab11A siRNA-treated Hep-2 cells were infected with C. trachomatis (MOI 3). At 44 h p.i., cells were lysed and infectious bacteria were titrated on HeLa cells. Numbers of infectious bacteria in KD cells were expressed as IFU (inclusion forming units). shRNA against Luciferase was used as a control. (A, C) Values were obtained from three independent experiments and are mean±SE. Student's t-test was performed to determine p-value, *<0.05. (0.37 MB TIF) [file ppat.1000615.s001.tif]

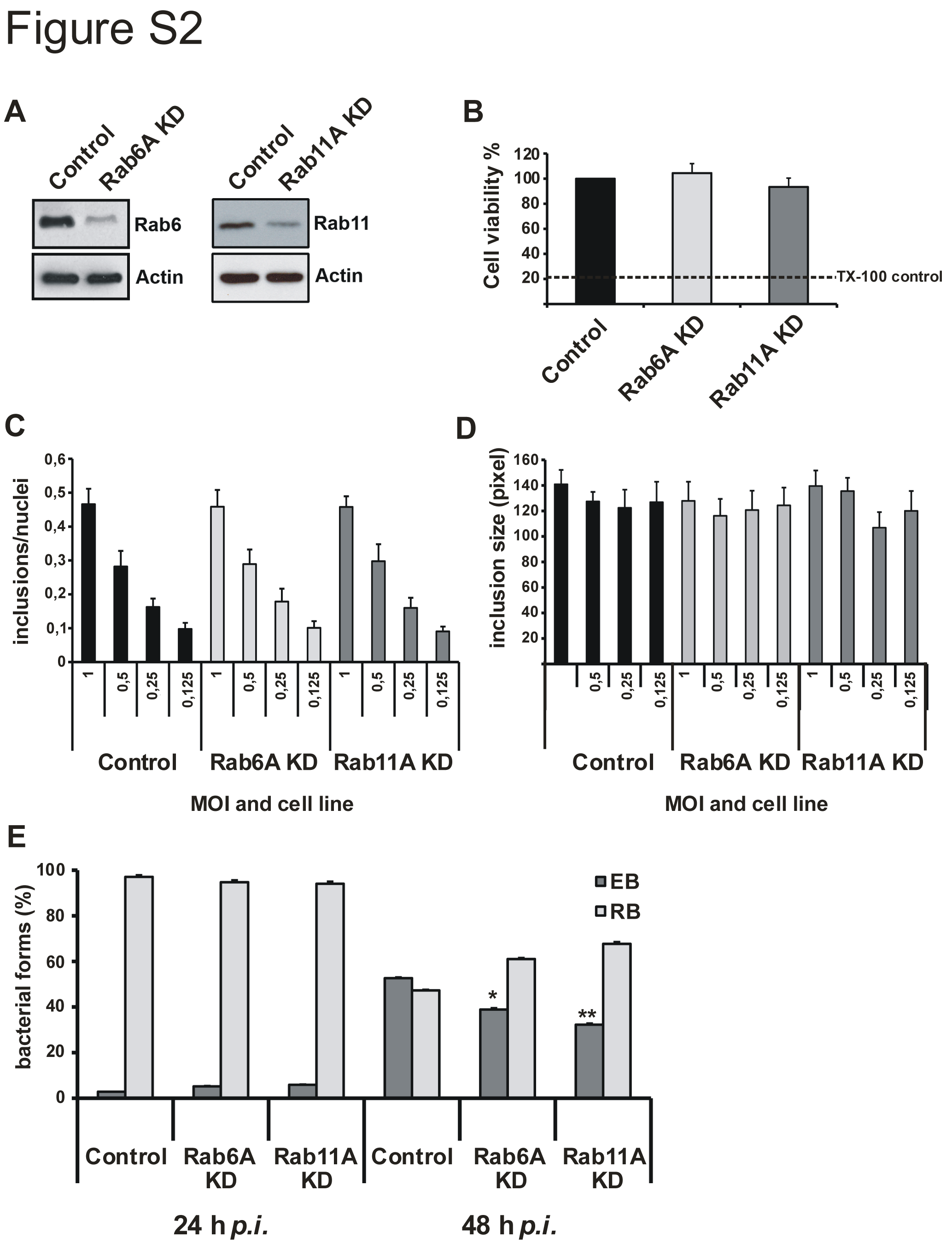

Supplement: Figure S2 — Chlamydia infection in stably expressing Rab protein knockdown HeLa cells. A) Control (Luciferase), Rab6 KD and Rab11 KD cells were lysed in RIPA buffer and proteins were separated by SDS-PAGE. Proteins were transferred to PVDF membranes followed by immunoblot with antibodies specific for Rab6, Rab11 and actin. Actin was used as loading control. Blots are representative of n = 3. B) Cell viability of stable KD cells monitored using the WST-1 assay. Diagram shows percent of cell viability normalized to luciferase control cells. TritonX-100 treatment prior to analysis was used as a negative control for the assay. C and D) Quantification of formed inclusions and analysis of inclusion size in KD and control cells. Cells were infected with different MOIs as indicated. At 24 h p.i., cells were fixed and stained for chlamydial MOMP. DNA was counter stained with Hoechst reagent. Samples were analysed with an automated microscope. D) Inclusion size was measured by determination of the pixel area. E) Quantitative analysis of EBs and RBs from electron micrographs. KD cells were infected with C. trachomatis for 24 h and 48 h. Luciferase cells were used as a control. Relative numbers of EBs and RBs, expressed as a percentage, were determined in more than 33 cells. (B–E) Values were obtained from three independent experiments and are mean±SE. Student's t-test was performed to determine p-value, *<0.05, **<0.01. (0.59 MB TIF) [file ppat.1000615.s002.tif]

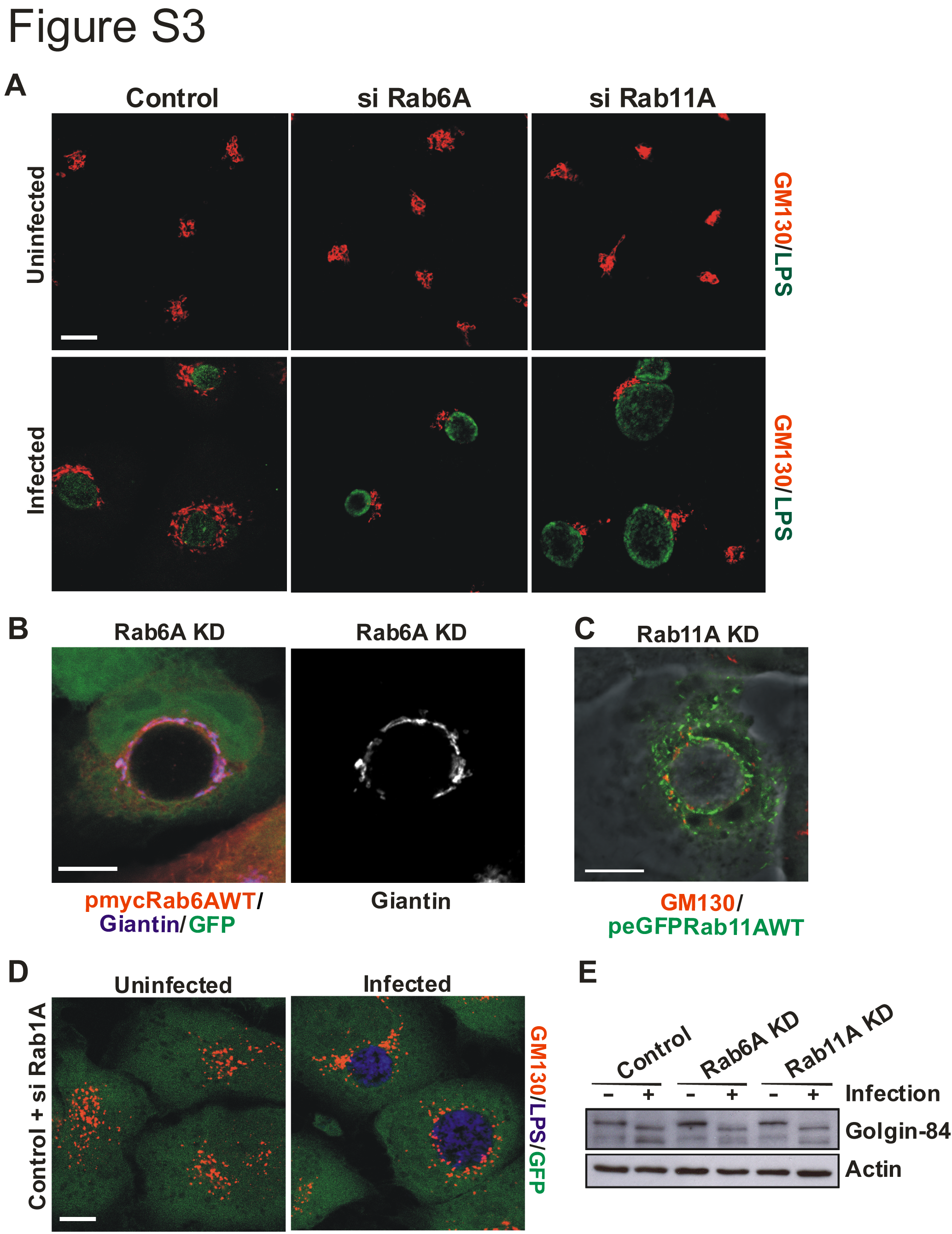

Supplement: Figure S3 — A) siRNA-treated cells were infected with C. trachomatis (MOI 1) for 24 h. Cells were then fixed and stained with an antibody specific for the Golgi protein GM130 (red channel) and chlamydial LPS (green channel). Samples were analysed at a LSCM. Overlays are shown. B) Expression of pmyc∶Rab6AWT in Rab6A KD cells. At 26 h p.i., cells were fixed and stained with antibodies specific for myc (red channel) and the Golgi protein Giantin (blue channel). Samples were analysed at a LSCM. Merge picture is shown. A single image for Giantin staining is shown in gray scale. C) Expression of peGFPRab11AWT in Rab11A KD cells. At 26 h p.i., cells were fixed and stained with an antibody specific for the Golgi protein GM130 (red channel). Stable KD cells express eGFP as a marker for shRNA expression, therefore only high levels of eGFP-Rab11 could be detected. Intensity of the GFP channel was reduced to better visualize eGFP-Rab11. Samples were analysed at a LSCM. Merge pictures are shown. D) Rab1A-siRNA-treated Luciferase control cells were infected with C. trachomatis (MOI 1) for 24 h. Cells were then fixed and stained with an antibody specific for the Golgi protein GM130 (red channel) and chlamydial LPS (blue channel). Stable KD cells express GFP as a marker of shRNA expression. Samples were analysed at a LSCM. Overlays are shown. E) Golgin-84 cleavage in KD cells. Control (Luciferase), Rab6A KD and Rab11A KD cells were lysed in RIPA buffer and proteins were separated by SDS-PAGE. Proteins were transferred to PVDF membranes followed by immunoblot with antibodies specific for golgin-84 and actin. Actin was used as loading control. Images and blots are representative of n = 3. Scale bar, 10 µm. (3.13 MB TIF) [file ppat.1000615.s003.tif]

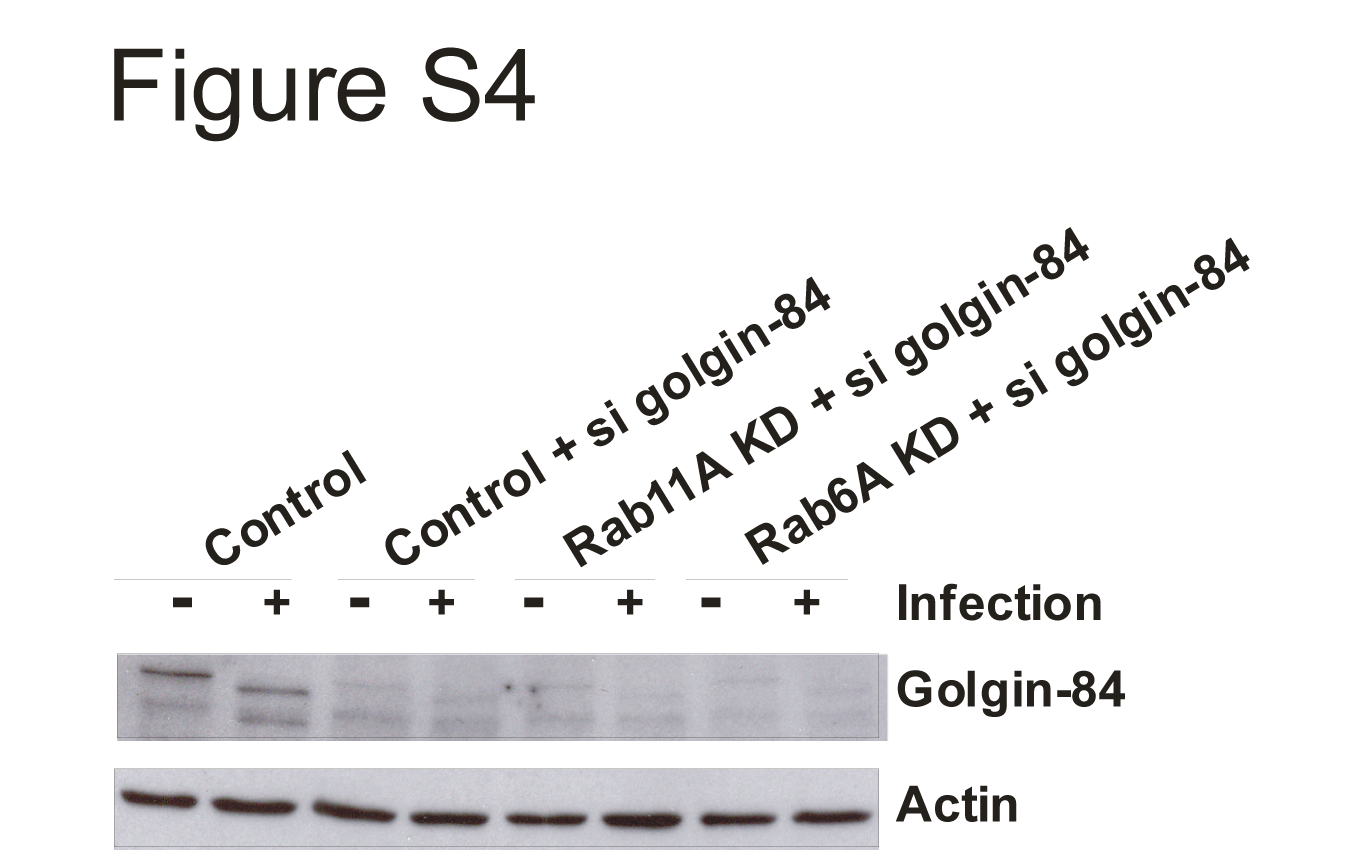

Supplement: Figure S4 — KD of golgin-84 in transiently transfected Rab6A and Rab11A KD cells. Golgin-84 KD efficiency in KD cells. Transiently transfected control (Luciferase), Rab6A and Rab11A KD cells were lysed in RIPA buffer and proteins were separated by SDS-PAGE. Proteins were transferred to PVDF membranes followed by immunoblot with antibodies specific for golgin-84 and actin. Actin was used as loading control. Blot is representative of n = 3. (0.36 MB TIF) [file ppat.1000615.s004.tif]

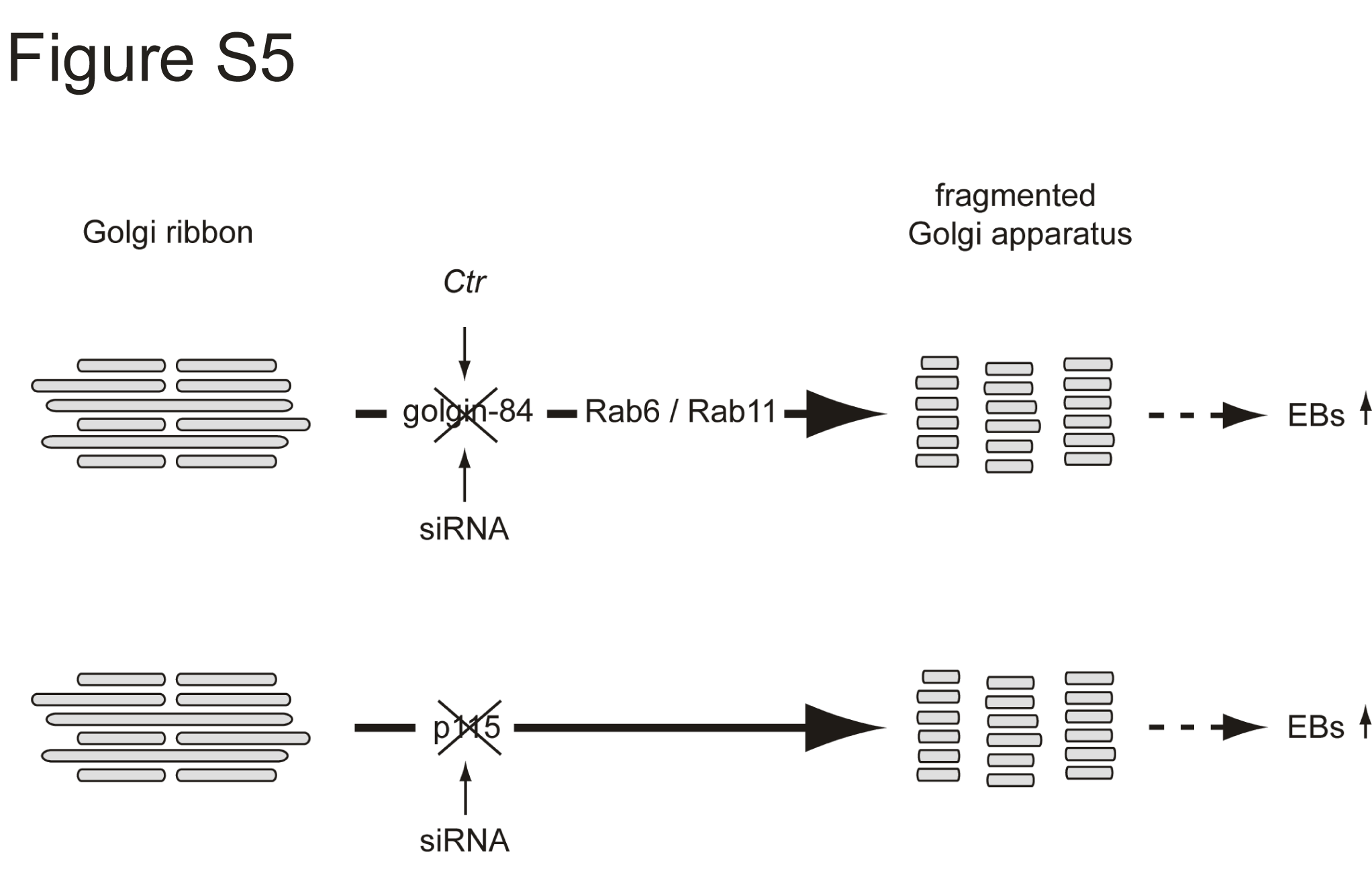

Supplement: Figure S5 — Schematic model of Rab regulated Golgi fragmentation and Chlamydia development. Under normal cellular conditions Golgi matrix proteins, including golgin-84, stabilize the GA, whereas Rab6 and Rab11 stimulate GA fragmentation. Upon infection, Chlamydia can influence these factors, either positively or negatively, to ensure efficient development. In this model C. trachomatis targets golgin-84, Rab6 and/or Rab11 to destabilize Golgi structure and stimulate ceramide acquisition, thus enhancing the development of infectious particles. (0.22 MB TIF) [file ppat.1000615.s005.tif]
